# Characterization of the Tumor Microenvironment and Tumor–Stroma Interaction by Non-invasive Preclinical Imaging

**DOI:** 10.3389/fonc.2017.00003

**Published:** 2017-01-31

**Authors:** Nirilanto Ramamonjisoa, Ellen Ackerstaff

**Affiliations:** ^1^Department of Medical Physics, Memorial Sloan Kettering Cancer Center, New York, NY, USA

**Keywords:** cancer, microenvironment, stroma, metabolic cooperation, tumor–stroma interaction, preclinical multimodal imaging

## Abstract

Tumors are often characterized by hypoxia, vascular abnormalities, low extracellular pH, increased interstitial fluid pressure, altered choline-phospholipid metabolism, and aerobic glycolysis (Warburg effect). The impact of these tumor characteristics has been investigated extensively in the context of tumor development, progression, and treatment response, resulting in a number of non-invasive imaging biomarkers. More recent evidence suggests that cancer cells undergo metabolic reprograming, beyond aerobic glycolysis, in the course of tumor development and progression. The resulting altered metabolic content in tumors has the ability to affect cell signaling and block cellular differentiation. Additional emerging evidence reveals that the interaction between tumor and stroma cells can alter tumor metabolism (leading to metabolic reprograming) as well as tumor growth and vascular features. This review will summarize previous and current preclinical, non-invasive, multimodal imaging efforts to characterize the tumor microenvironment, including its stromal components and understand tumor–stroma interaction in cancer development, progression, and treatment response.

## Introduction—The Tumor Microenvironment (TME)

**Figure 1:**
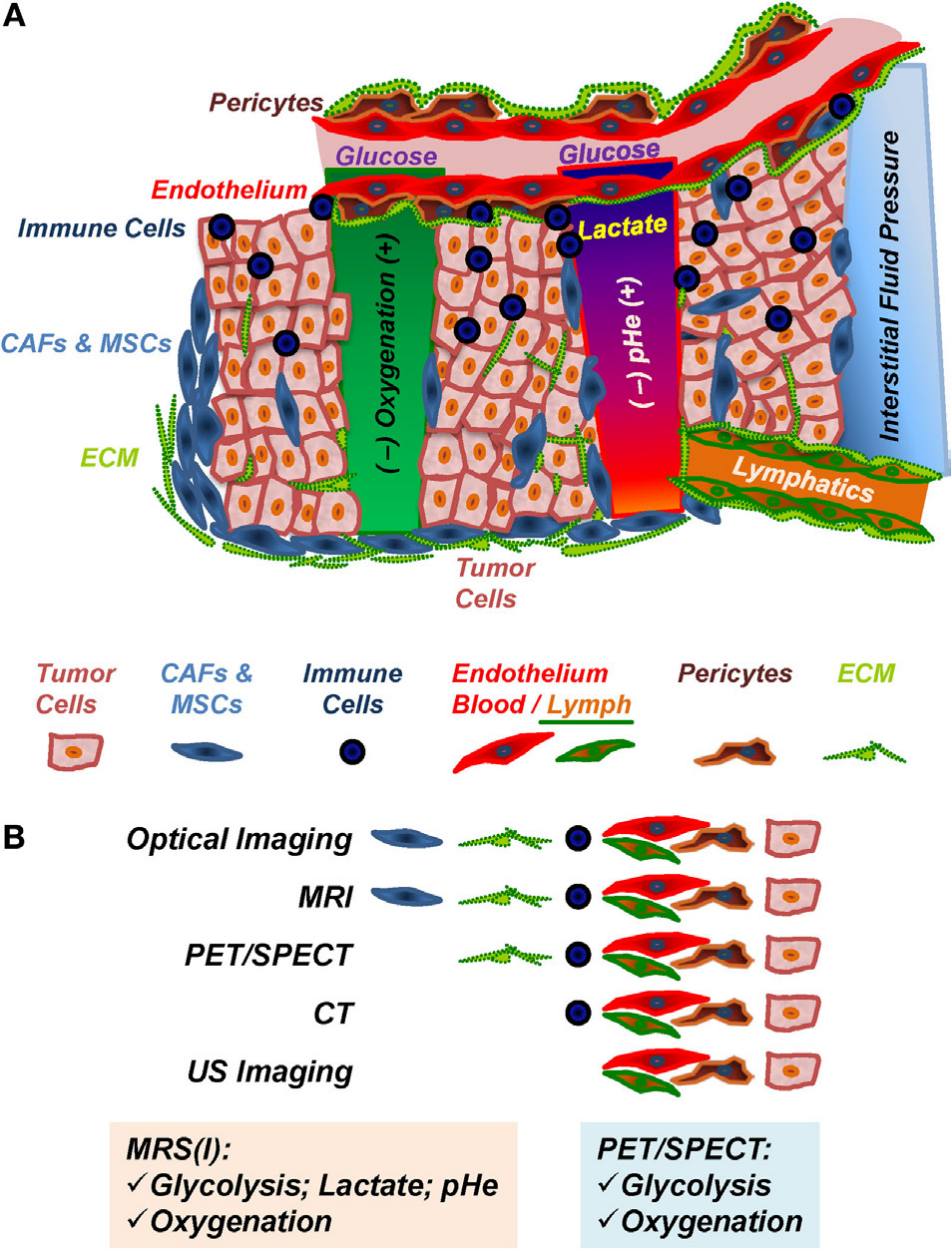
**The tumor microenvironment (TME)**. **(A)** Components and *in vivo* imaging of the TME. Immune cells include tumor-associated macrophages, antigen-presenting cells, myeloid-derived suppressor cells, and lymphocytes; CAFs, cancer-associated fibroblasts; MSCs, mesenchymal stem cells; ECM, extracellular matrix, consisting of collagens, laminins, and other matrix proteins, which is remodeled by ECM-degrading proteases; endothelial cells, pericytes, and vascular ECM compose the tumor blood and lymph vasculature. **(B)** Preclinical *in vivo* imaging of the TME. MRI, magnetic resonance imaging; PET, positron emission tomography; SPECT, single photon emission computer tomography; CT, computer tomography; US, ultrasound.

The TME (Figure 1A), composed of tumor cells and stroma, is often characterized by hypoxia, vascular abnormalities, low extracellular pH (pHe), increased interstitial fluid pressure (1–7), increased aerobic glycolysis (Warburg effect) (8, 9), glutamine addiction (10–13), and altered choline-phospholipid metabolism (14–19). Recent evidence suggests that metabolic reprograming in the course of tumor development and progression increases in more aggressive cancer cells/tumors the ability to easily adapt metabolism to the most advantageous pathways, beyond the Warburg effect, in order to ensure their growth and survival in response to varying environmental stimuli, such as hypoxia or limited nutrient supply (20–24). Altered metabolic content in tumors may affect cell signaling and degree of cellular differentiation (11, 25–27).

**Figure 2:**
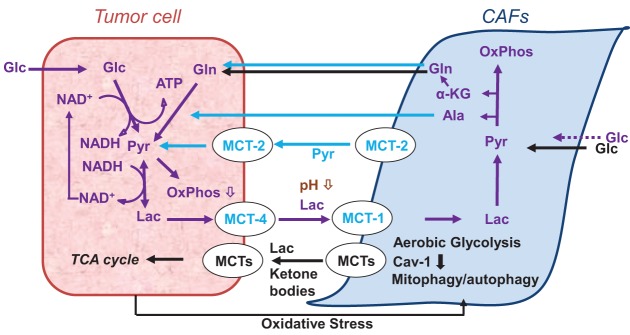
**Models of cancer cell–cancer-associated fibroblast (CAF) metabolic cooperation in the tumor microenvironment, promoting survival, growth, and metastases (38, 39)**.

While previous research focused extensively on the tumor cells, over the last two decades or so, further evidence emerged that the tumor stroma is altered during tumor development/progression and that the tumor–stroma interaction plays an essential role in tumor metabolism (Figure 2), development, progression, and treatment response (2, 22, 23, 26, 28–37).

The stroma in solid tumors consists of extracellular matrix (ECM), and stromal cells, including fibroblasts, endothelial cells, pericytes, and various immune cells, such as macrophages, neutrophils, mast cells, myeloid progenitors, and lymphocytes (Figure 1A), with cancer cells playing an active role in the recruitment and metabolic reprograming of stromal cells (Figure 2) (22, 26, 40) and the dynamic remodeling of ECM by tumor and stromal cells promoting tumor progression (41–44).

**Figure 3:**
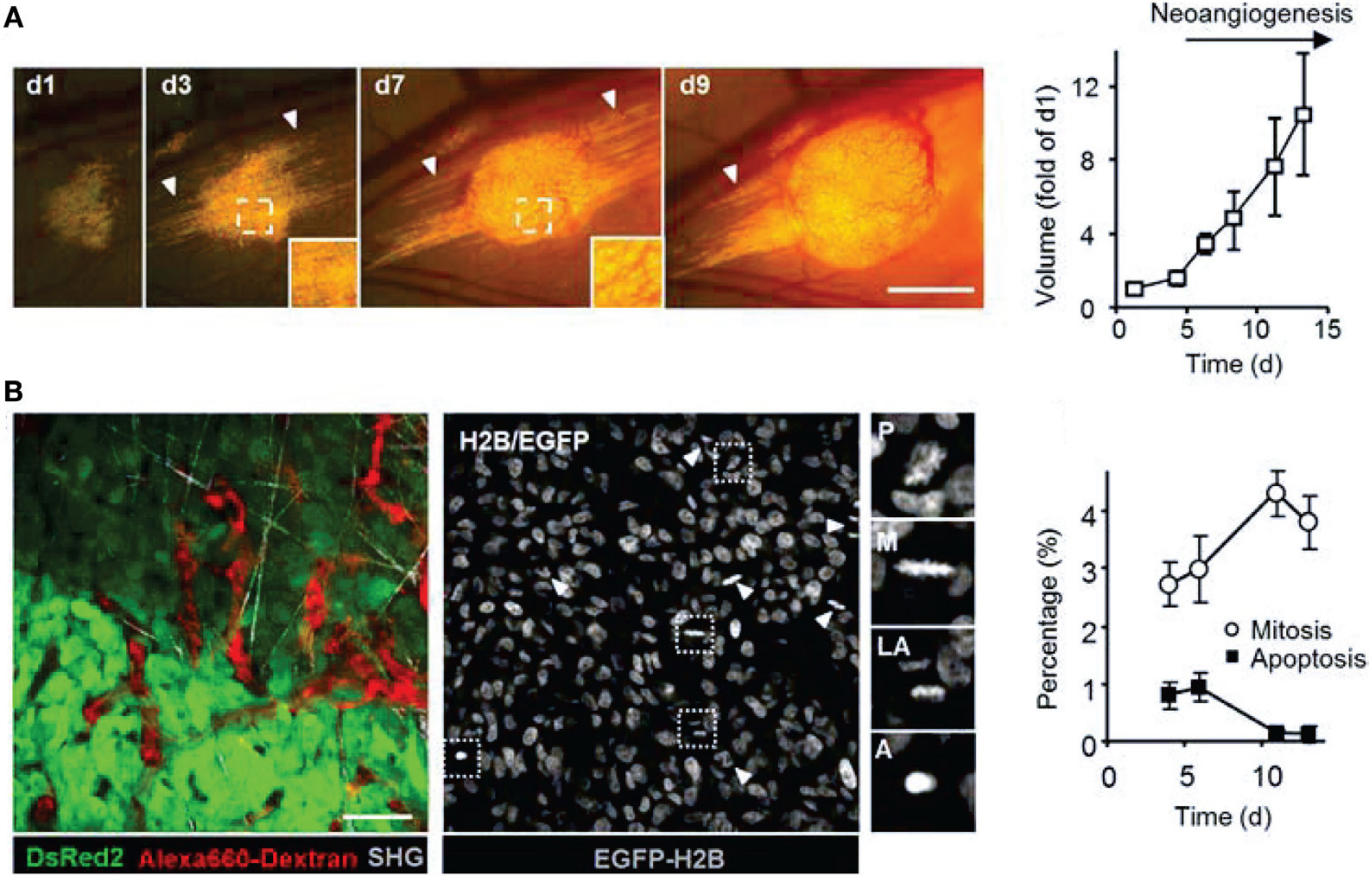
**Intravital microscopy of the tumor microenvironment**. **(A)** Epifluorescence microscopy was used to monitor and quantify tumor growth in a human fibrosarcoma xenograft model. The invasion of tumor into the surrounding tissue during growth can be visualized (white arrowheads). Bar 50 µm. Adapted with permission from Ref. (66). **(B)** Tumor morphology, vascularization, proliferation, and apoptosis in a human fibrosarcoma xenograft, as detected by intravital microscopy: tumor cells express cytoplasmic DsRed2 and nuclear histone 2B (H2B)-EGFP. Collagen fibers are detected by second-harmonic generation. Non-disrupted vessels are detected from the fluorescence signal of i.v.-administered Alexa660-Dextran. Bar 50 µm. Nuclear morphology including mitotic (white arrowheads) and apoptotic figures (black arrowhead) can be derived and quantified from imaging H2B-EGFP and DsRed2. Insets show prophase (P), metaphase (M), late anaphase (LA), and apoptotis (A). Bar 50 µm. Adapted with permission from Ref. (66).

**Figure 4:**
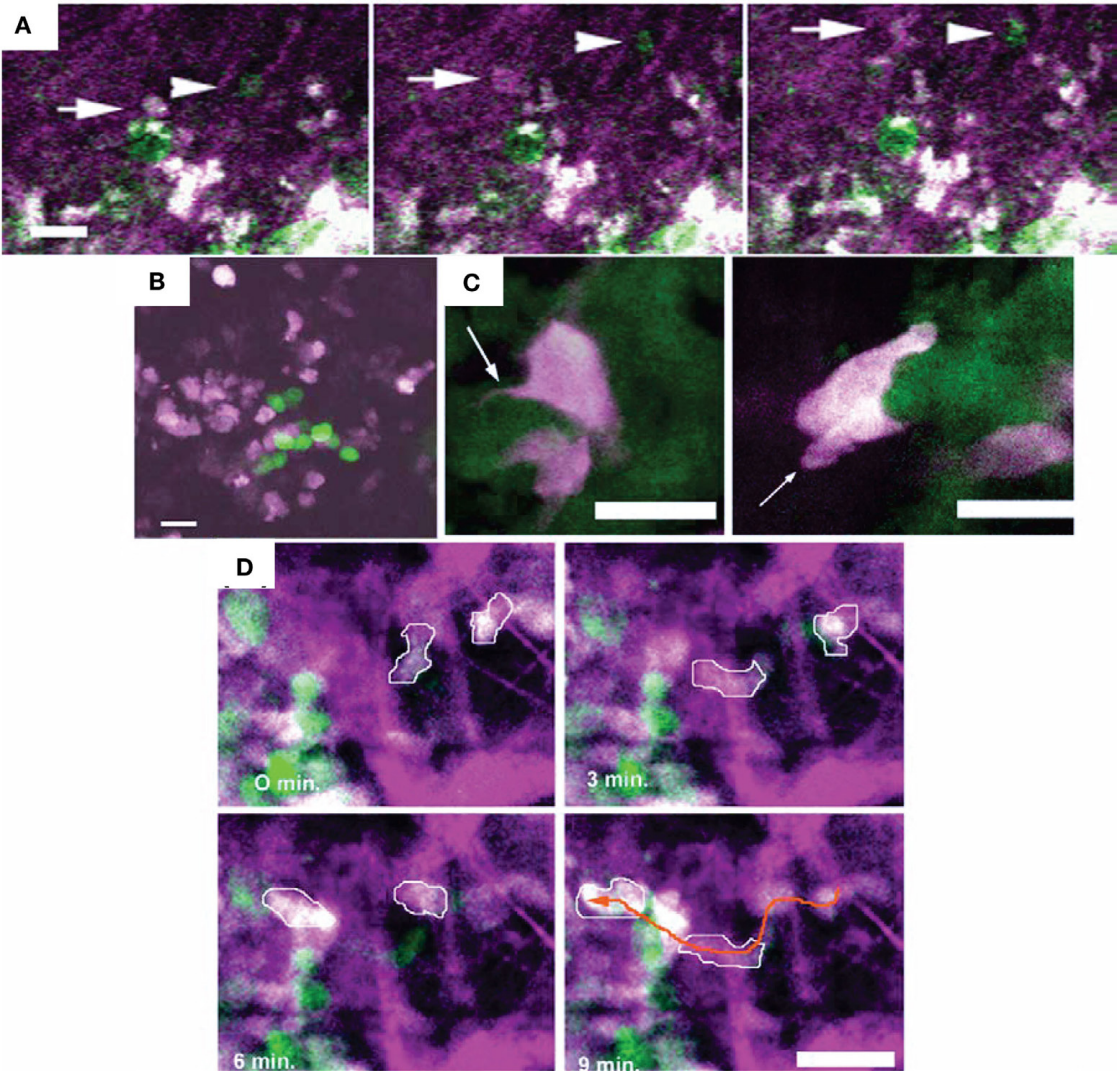
**Different motility and invasion of low-metastatic, GFP-expressing (green) and high-metastatic, CFP-expressing (white) mammary tumor cells within the collagen network (purple) was imaged by *in vivo* intravital microscopy with FL and second-harmonic generation**. Bars 25 µm. **(A)** Time series demonstrating the migration of GFP-expressing (arrow head) and CFP-expressing (arrow) tumor cells along collagen fibers. **(B)** Metastatic growth of color-coded cells in the lung. **(C)** Protruding filopod (arrow, left) and lamellapod (arrow, right) of CFP-expressing cell near GFP-expressing cells. **(D)** Overall, the high-metastatic cells (outlined in white) move more frequently (see orange arrow path) than the low-metastatic cells (green). Adapted with permission from Ref. (67).

**Figure 5:**
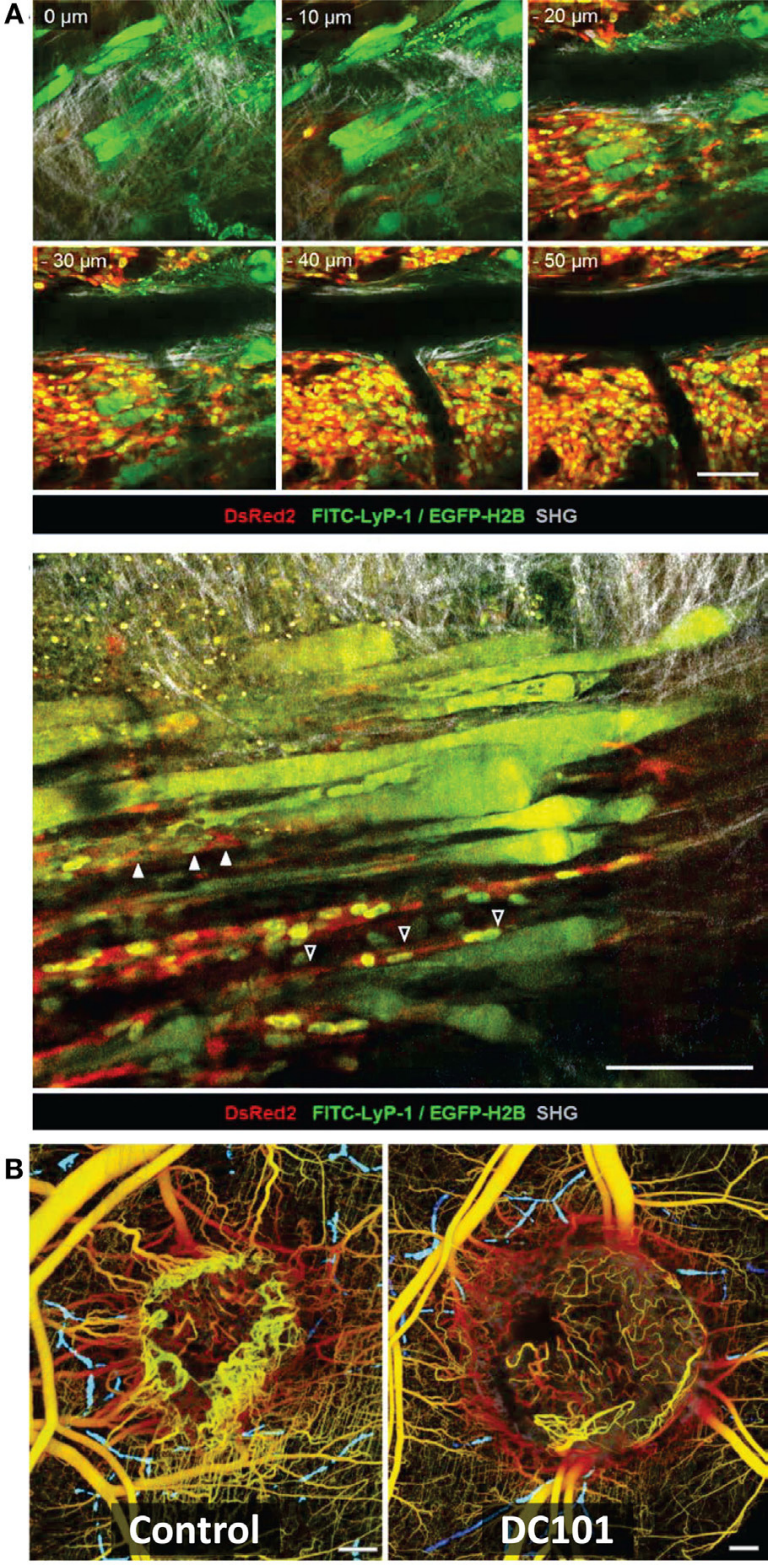
**Intravital microscopy of the tumor blood vessels, lymph vasculature, and vascular response to treatment**. **(A)** Top: Z-stack of lymphatics detected by near-infrared fluorescence (NIRF) multiphoton microscopy of FITC-tagged LyP-1 peptide (green), collagen fibers detected by second-harmonic generation, and tumor cells imaged by epifluorescence of cytoplasmic DsRed2 (red) and nuclear histone 2B (H2B)-EGFP (green) shows lymph vessels at the tumor margin. Bottom: intralymphatic (white arrowheads) and perilymphatic (black arrowheads) invasion of fibrosarcoma cells expressing cytoplasmic DsRed2 (red) and H2B-EGFP (green). Bars 100 µm. Adapted with permission from Ref. (66). **(B)** *In vivo* optical frequency domain imaging of blood [depth denoted from red (up to 2 mm deep) to yellow (superficial)] and lymph (blue) vessels in control and DC101-treated tumors, depicting the antivascular effect of VEGFR-2 inhibition. Adapted with permission from Ref. (68).

**Figure 6:**
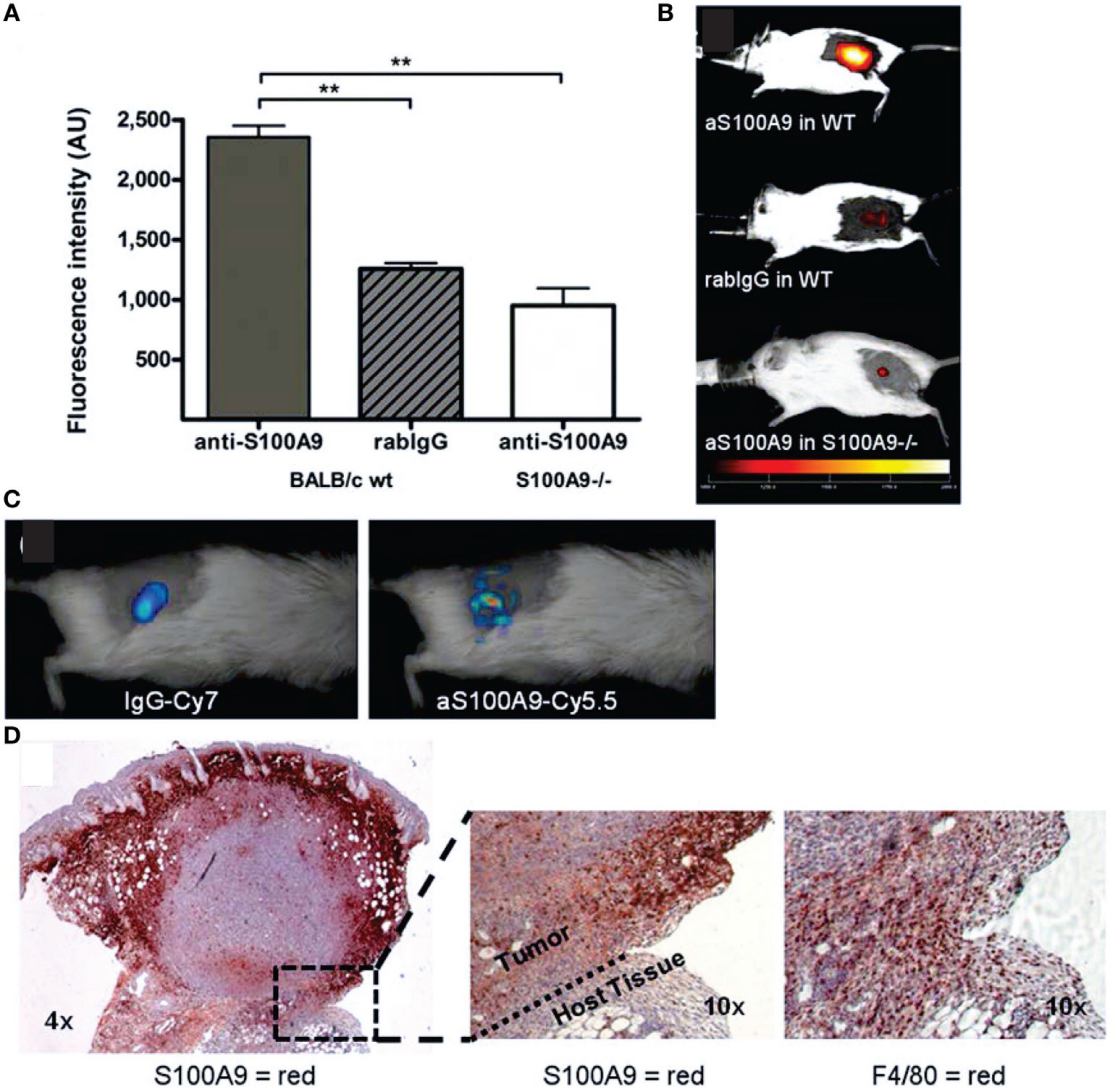
***In vivo*** immune cell imaging. **(A,B)** Specificity of *in vivo* imaging of immune cells in 4T1 mammary breast tumors by fluorescence-reflectance imaging with a Cy5.5-labeled polyclonal antibody against murine S100A9 (aS100A9-C5.5). **(C)** Fluorescence molecular tomography of coinjected rabIgG-Cy7 and aS100A9-C5.5 demonstrates homogeneous perfusion (left) and immune cell distribution (right), respectively. **(D)** *Ex vivo* validation shoe S100A^+^ cells in the tumor periphery corresponding to F4/80^+^ TAMs. Adapted with permission from Ref. (69) © by the Society of Nuclear Medicine and Molecular Imaging, Inc.

**Figure 7:**
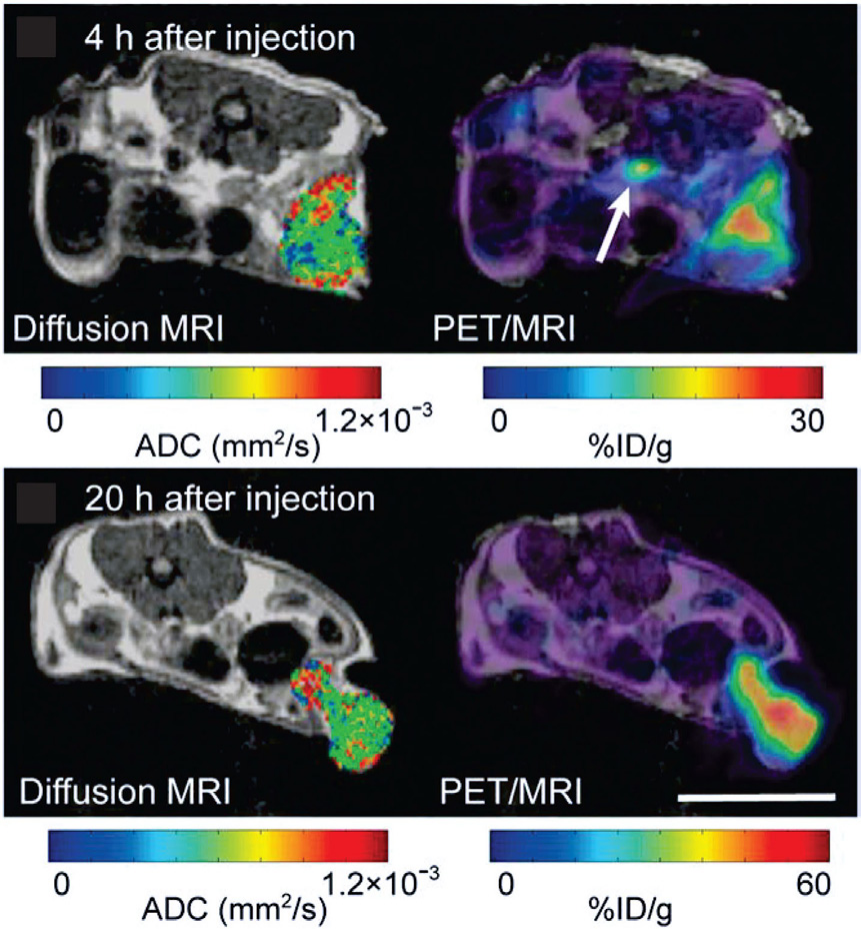
**In a carcinoembryonic antigen (CEA)-expressing colorectal adenocarcinoma model, simultaneous magnetic resonance imaging (MRI)/positron emission tomography (PET) at 4 h and 20 h after the injection of a radiolabeled antibody against CEA (^64^Cu-DOTA-NHS-M5A) demonstrates its accumulation in the tumor over time, while the apparent diffusion coefficient across the tumor remained largely unchanged**. Adapted with permission from Ref. (70) © by the Society of Nuclear Medicine and Molecular Imaging, Inc.

Multiple preclinical imaging techniques (Table 1; Figure 1B) have been developed to visualize and quantify specific characteristics of the TME (5, 45, 46). This review summarizes the efforts to image and characterize non-invasively the TME (Figure 1), including its stromal components, and tumor–stroma interaction (Figures 2–7) in preclinical cancer. Stromal components and their imaging are described in the context of preclinical cancer in Section “The Tumor Stroma and Its Imaging.” Section “Non-invasive Multimodal Imaging of Tumor–Stroma Interaction” focuses on the more recent attempts to assess the interaction of stromal components with cancer cells by non- or minimally invasive preclinical multimodal imaging.

**Table 1:** **Summary of modalities for *in vivo* imaging of the tumor microenvironment in preclinical (small animal) tumor models**.

Imaging modality			Resolution			Contrast agent
			In-plane	Coverage/depths	Temporal per frame	
Optical	Bioluminescence imaging (47, 48)	BLI	>3–5 μm	1–2 cm	>1 s to min	Reporter genes
	Fluorescence imaging (47, 48)	FLI	2–3 µm	<1 cm	>1 s to min	Fluorophores, fluorescent nanoparticles
	Fluorescence lifetime microscopy (49)	FLIM	nm range	~1,000 μm	>1 s to min	Fluorophores, fluorescent nanoparticles
	Fluorescence micro-lymphangiography (50)	FML	50 µm	200 µm	Video rates	FITC-dextran
	Fluorescence molecular tomography (51, 52)	FMT	<1 mm	1–2 mm	>1 s to min	NIRF dyes, quantum dots, reporter genes
	Fourier transform infrared imaging (53–56)	FTIR	>~3–5 μm	<20 μm	>1 ms to min	Endogenous
	Near-infrared fluorescence imaging (50)	NIRF	~200 μm	<3–4 cm	50–800 ms	NIRF dyes, quantum dots, reporter genes
	Optical coherence tomography (57)	OCT	<7.5 μm	2–3 mm	<1 s	Endogenous
	Photoacoustic imaging (tomography) (52, 58–60)	PAI (PAT)	100 µm	<5–6 cm	>1 s to min	Fluorophores, nanoparticles, quantum dots
	Second-harmonic generation microscopy (61, 62)	SHG	<1 μm	≤1 mm	>10 s	Endogenous
	Third-harmonic generation microscopy (61)	THG	<1 μm	≤1 mm	>10 s	Endogenous
X-rays	Computer tomography (47, 48, 50, 52)	CT	~50–200 μm	Whole body	>20 s	Water-soluble, iodinated probes
Magnetic resonance	Magnetic resonance imaging (47, 48, 50, 52, 63)	MRI	~25–100 μm	Whole body	>2 min	Label-free
			0.1–0.3 mm	Whole body	min to h	Gd- or iron-oxide-based probes; dendrimer-based macromolecules
	Magnetic resonance spectroscopic imaging (64)	MRSI	mm range	Whole body	min to h	Endogenous; injected marker or metabolic substrates
	Electron paramagnetic resonance imaging (65)	EPR	>0.5 mm	cm	min to h	Injected tracer
Nuclear	Positron emission tomography (47, 48, 52)	PET	1–2 mm	Whole body	>10 s to min	Radiolabeled substrates (nutrients, antibodies, antibody fragments), activatable probes
	Single photon emission computer tomography (47, 52)	SPECT	1–2 mm	Whole body	min	Radiolabeled antibodies, antibody fragments, and antigens
Ultrasound	Ultrasound imaging (47, 52)	US	50–500 µm	mm to cm	>1 s to min	Endogenous; targeted microbubbles

*Imaging modalities are color-coded separating optical, X-ray, magnetic resonance-, nuclear-(radioactivity-), and ultrasound-based imaging methods*.

## The Tumor Stroma and Its Imaging

In this chapter, we describe briefly the stromal components and their imaging with its strengths and limitations.

### The ECM

The ECM, a complex structure composed of laminins, collagens, proteoglycans, fibronectin, elastin, etc. (71), changes its composition during cancer progression (41, 72, 73). Many of its components are regulated by matrix metalloproteinases (MMPs) which are involved in growth signaling [by proteolytic activation of the transforming growth factor-β (TGF-β) pathway], apoptosis, and angiogenesis (73–76).

Available imaging methods focus on targeting the ECM component itself or the enzymes that degrade it, typically, by using activatable imaging probes (Figure 1B; Table 1). Imaging of cell–matrix adhesion can elucidate the dynamic interplay of cells and surrounding tissue during ECM remodeling, immune cell recruitment, wound healing, and cancer metastasis (77).

#### Collagen Imaging

Methods, such as colorimetry (78), weight measurements (79), atomic force microscopy (80–82), and immunostaining (83–85), to image collagen structures risk their destruction and are limited by their *in vivo* translatability. The dorsal skinfold (window) chamber setup allows optical measurements by replacing skin with glass but may lead to collagen structural changes due to inflammation and mechanotransduction by the glass (86). The advances in ultrafast optics significantly improved the ability to image fibrillar collagen (the predominant structural protein in mammalian ECM and mostly type I) by second-harmonic generation (SHG) or third-harmonic generation (61) microscopy *in vivo* and *ex vivo* (87–91). The strength of SHG imaging is its specificity to fibrillar collagen (62, 87, 89, 92) and that it can be fairly easily combined with other optical imaging methods, *in vivo* (Figures 3–5A) and *ex vivo* (49, 90, 93–95). Ability for clinical translation has been demonstrated in breast cancer patients by combining SHG and bright-field high-resolution microscopy with large field of view to design a semi-automated technique to predict survival based on collagen fiber classifications (93). Recently, confocal microscopy has been used *in vivo* to detect collagen turnover after introduction of fluorescent fibrillar collagen into the dermis of live mice (96). However, all optical imaging methods suffer from their limited imaging depth, rendering them often an invasive tool and limiting their clinical translation (49, 57). Thus, the diagnosis and treatment of pathologies related to collagen remodeling has benefited greatly from the development of collagen-binding or hybridizing peptides, bearing an imaging contrast agent (CA) for, e.g., magnetic resonance imaging (MRI) or fluorescence imaging, or theranostic agents, to image triple-helical, intact, and/or unfolded, denatured collagen and treatment response (97). Other imaging modalities [e.g., ultrasound (US) (98, 99), optical coherence tomography (OCT) (100, 101), Fourier transform infrared spectroscopic imaging (53), or multispectral photoacoustic imaging (PAI) (102)], and various collagen-targeted agents, e.g., quantum dots (84, 85, 103, 104) or collagen-mimetic peptide-based imaging agents (105, 106) are being developed/applied to improve collagen imaging and to measure collagen turnover during tissue remodeling.

#### MMP Imaging

The key role that various MMPs play during cancer initiation and progression, with clear links to tumor invasion and metastasis (107), make them desirable treatment and cancer imaging targets (108). Targeted probes to image MMPs *in vivo* by optical imaging (fluorescence and bioluminescence), positron emission tomography (PET), single photon emission computer tomography (SPECT), and MRI have been developed (108–110), with a “broad-spectrum” MMP-activatable fluorescence probe available commercially (MMPSense, PerkinElmer, Akron, OH, USA). Each modality and imaging probe displays strengths and weaknesses in effectively imaging MMP activity (108, 110). However, optical imaging has limited penetration depth (108) and, while tomography is possible, anatomical information is lacking. Targeted, inhibitor-based PET and SPECT probes harness the excellent sensitivity of radioactive tracers and may be theranostic, but their synthesis may be difficult and, so far, it has not been possible to quantify proteolytic activity *in vivo* due to non-specific binding (108, 109, 111). Recently, an ^18^F-labeled MMP-activatable PET probe has been developed to overcome the lack of specificity of inhibitor-based probes (112). Photoacoustic tomography (PAT or PAI) (58, 113, 114) combines ultrasonic resolution with electromagnetic-enhanced contrast to obtain quantitative information on tissue structure, blood flow, and perfusion, and, through targeted probes (59), on receptor status or enzyme activity. For example, a photoacoustic probe activated by MMP-2 and MMP-9 demonstrated sensing of MMP-2/-9 activity in a follicular thyroid carcinoma model (115). With an imaging depth of >30 mm, depending on setup and desired spatial resolution (58), PAT expands on the tissue penetration of up to 20 mm typical for optical imaging (51, 108) and is thus suitable to monitor non-invasively tumor characteristics in orthotopic preclinical cancer models. The lack of anatomical/morphological information inherent to optical imaging, PAT/PAI, and PET/SPECT can be overcome by multimodal imaging. Using fluorescence molecular tomography (FMT) (51) coregistered with MRI, Salaun et al. (116) found increasing MMP-13 levels as lung tumors progressed. In skin squamous cell carcinoma xenografts, MMP-2, -3, -7, -9, -12, and -13 activities correlated with degree of angiogenesis and tumor invasion, as imaged by FMT combined with μCT (117). PAT/PAI, combined with US and OCT, can provide non-invasively morphological and functional tumor characteristics (60). Molecular MRI to measure MMP activity using protease-modulated CAs is still emerging and is hampered by its insensitivity, requiring long acquisition times but is potentially quantitative, and anatomical information can be obtained in the same setting (108, 109). Of note is that the interpretation of MMP images obtained with the typically broad-spectrum probes (110) is further complicated by the function of MMPs in biological processes beyond ECM remodeling (111).

#### Proteoglycan (Hyaluronan) Imaging

Another major ECM constitutent, the proteoglycan hyaluronan [hyaluronic acid (HA), hyaluronate] is a high molecular weight glycosaminoglycan with a significant role in tumor growth and metastasis (118, 119), acting as tumor suppressor or promoter depending on its molecular weight (120).

It is degraded by hyaluronidases (Hyals), with hyaluronidase-1, -2 (Hyal1, Hyal2) currently being the most studied in cancer (119). Hyal1 overexpression has been associated with more aggressive tumors in a variety of epithelial cancers (e.g., bladder, colorectal, breast, and ovary), while Hyal2 may function as a tumor suppressor or promoter (119). The development of various HA probes to image HA turnover and clearance and of theranostic HA probes where encapsulated drugs are released in response to Hyal activity (119, 121, 122) has expanded greatly in recent years, and a detailed review is beyond the scope of this paper. HA probes often exploit the high specificity of HA for the CD44 receptor, a transmembrane receptor overexpressed in many tumor cell types (120, 121, 123–134). Single moiety, HA-based CAs have been used to image Hyals activity by MRI (135) and NIRF (124). Fluorescence correlation spectroscopy and Forster resonance energy transfer of HA-conjugated probes have shown promise in quantitative bladder cancer staging by detecting shedded Hyals in urine samples (119). Also, fluorescent HA probes may be used in an intraoperative to assess Hyal activity or drug delivery of theranostic probes (119). Often, HA probes contain more than one CA moiety to harness the strength of multimodal imaging, such as MRI/optical imaging (123, 136), MRI/computer tomography (CT) (137), NIRF/CT (138), or NIRF/PA imaging (139–141) to improve diagnostic capability and monitoring of therapeutic efficacy (121, 142).

#### Other ECM Constitutents

Only a few studies report the *in vivo* imaging of other ECM constituents, such as fibronectin or laminins, in cancer. Fibronectin, whose expression increases with epithelial–mesenchymal transition, is typically targeted to image tumor-associated angiogenesis (143, 144). Laminins are a family of glycoproteins which interact with other ECM proteins, assuring the ECM organization, and are involved in cellular signal transduction pathways (145), cell adhesion, migration, and proliferation (146) and thus affect in cancer, tumor invasion, angiogenesis, and metastasis (145, 147). While laminins and their function have been studied extensively *in vitro* or *ex vivo* (29, 148–150), *in vivo* studies directly imaging laminins have been limited. Cuesta et al. developed a fluorescent trimerbody recognizing an angiogenesis-associated laminin epitope, accumulating in tumors (151). Other studies have used imaging agents targeting laminin cell surface receptors directly or indirectly to detect or treat tumors (152–154).

### Mesenchymal Stromal (Stem) Cells (MSCs), Cancer-Associated Fibroblasts (CAFs), and Immune Cells

By tumor cells recruited adult, multipotent, non-hematopoietic stem cells (mesenchymal stromal (stem) cells, MSCs), typically derived from adipose tissue and bone marrow, have been found to differentiate into osteoblasts, CAFs, and pericytes among other cell types (155). In tumors, MSCs may contribute to tumor initiation, progression, angiogenesis, and metastasis, while also impacting immune function (155).

Cancer-associated fibroblasts are fibroblasts that reside within the tumor or tumor margins (156). They promote tumorigenic features, such as drug resistance, ECM modulation, chronic inflammation, and invasiveness (156). They may originate from normal fibroblasts or smooth muscle cells (altered by tumor cells), bone marrow-derived stem cells (mesenchymal stromal (stem) cells, MSCs), recruited and altered by tumor cells, or epithelial cells through transdifferentiation to myofibroblasts, or endothelial cells through endothelial-to-mesenchymal transition (156–158).

The TME (Figure 1) includes various immune cells: innate [tumor-associated macrophages (TAMs)], neutrophils, mast cells, myeloid-derived suppressor, dendritic, and natural killer cells, and adaptive (T and B lymphocytes), with TAMs and T cells the most prevalent cell types (159). Immune cells may enhance tumor growth and metastasis or exhibit antitumor immunity by modulating the immune and inflammatory milieu in the TME through paracrine and autocrine cell interactions, and thus, affecting the production of pro-angiogenic and growth factors, proteases, recruitment of other hematopoietic cells, or release of reactive oxygen or nitrogen species (159, 160). Immunotherapy aims at enhancing the antitumor activity of tumor-associated immune cells (161).

#### MSC Imaging

In preclinical cancer models, the preferential homing of *ex vivo* cultured MSCs to tumor tissue and metastasis has been imaged non-invasively *in vivo* after intravenous/arterial injection of MSCs, pre-(multi-)labeled with bioluminescence (162–167), fluorescence (168), MRI (169–171), PET (170–173), or SPECT (171, 174) imaging probes (175). The tumor effects and/or localization of MSCs, pre-labeled with an imaging probe and coinjected with tumor cells, have been monitored *in vivo* with MRI (176) and bioluminescence (177–180) imaging. The preferential accumulation of MSCs at sites of inflammation and tumors makes them an ideal vehicle for treatment delivery (155, 181, 182), and combined treatment/imaging MSC moieties are being developed for cell tracking and treatment monitoring (175, 177, 178, 183). While overall safe clinically (175, 184), *ex vivo* culture and pre-labeling of MSCs may lead to secondary tumors (185) and/or impact functionality (186). Using fluorescent, transgenic mice (187), potentially avoids *ex vivo* culture and pre-labeling of MSCs, with the disadvantage that typically all cell types express the imaging marker, limiting the *in vivo* identification of different stromal cell types, unless pre-labeled.

#### CAF Imaging

Various markers, such as α-smooth muscle actin (α-SMA), vimentin, and fibroblast-activation protein α (FAP), have been used to identify CAFs (188). In preclinical models, CAFs have been shown to promote breast tumor growth and metastasis by enhancing the recruitment of immune suppressor cells and TAMs (189) and to mediate collagen remodeling (190). The high expression of FAP in CAFs (191, 192) makes it a desirable target for diagnostic and therapeutic imaging, although it may also be expressed in some other tissues and tumor cells (192–194). One difficulty for the development of FAP-targeted *in vivo* imaging probes is that FAP shares peptide substrates with other post-prolyl peptidases, resulting in non-specific binding *in vivo* (192). Thus to track CAFs *in vivo* by MRI or NIRF imaging, Granot et al. took advantage of caveolae-mediated endocytosis in fibroblasts by pre-labeling CAFs *in vitro* with the CAs biotin-bovine serum albumin-gadolinium diethylenetriaminepentaacetic acid, Feridex, or 1,1′-dioctadecyl-3,3,3-tetramethylindotricarbocyanine iodide (195, 196). Recently developed FAP-specific, activatable NIR fluorescence probes (193, 194) show promise in *in vivo* imaging of FAP-expressing tumors. And novel cancer treatments based on the depletion of FAP-expressing stromal cells would greatly benefit from monitoring response *in vivo* with improved multimodal imaging probes (197).

#### Immune Cell Imaging and Monitoring of Immunotherapy

Multiple recent reviews summarize the imaging techniques applied to track various immune cell types *in vivo* and to monitor immunotherapy response, including the ability to optimize administration route of therapeutic immune cells (45, 47, 61, 63, 198–202).

To visualize immune cells *in vivo*, cells may be labeled *ex vivo* with paramagnetic, fluorescent, or radiochemical probes for MRI, FLI, or PET/SPECT respectively or transfected with reporter genes for PET, SPECT, bioluminescence imaging (BLI), and/or FLI (or FMT) before injection into the host (45, 198, 201, 203–206). Tumor-infiltrating lymphocytes, dendritic cells (DCs), or TAMs can be imaged by FLI or PET/SPECT, using fluorescently labeled (Figure 6) or radiolabeled antibodies or antibody fragments targeting cell-type specific surface receptors (198, 199, 201). Delivery of full-size antibodies may be affected by vascular dysfunction in tumors, thus, potentially affecting image quality and interpretation [false positives, reduced specificity (50)]. Targeted US microbubbles have been used to track B7-H3 expressing TAMs and tumor cells (198). The high endocytic activity of TAMs facilitates their imaging by MRI, PET, and to a lesser extent by CT, FLI, US, Raman imaging, or PAI, using nanoparticles, either uncoated (e.g., ultra-small superparamagnetic iron-oxide nanoparticles for MRI) or coated to increase macrophage affinity (199, 200). Nanoparticle uptake may vary across different macrophage populations and may be detected also in other cell types (e.g., tumor cells) to a variable degree, confounding potentially macrophage tracking, but has been used successfully in clinical lymph node cancer staging by MRI (200). Cellular MRI of DC migration using CAs (iron oxide-based nanoparticles, perfluorocarbon emulsions) permits repeated monitoring (a limitation in PET/SPECT) and does not require pre-labeling with reporter genes, as in BLI or FLI, but has lower sensitivity (63). Intravital microscopy of stroma–tumor cell dynamics (207) has been an essential tool in assessing lymphocytic interactions in tumors and draining lymph nodes by optical imaging (208). The clinical translatability of these imaging methods has been reviewed previously (202, 209), with optical methods currently limited to intraoperative imaging (50).

### Tumor Vasculature and Lymphatics—Endothelial Cells and Pericytes

Endothelial cells line the inside of tumor blood vessels and lymphatics and interact with pericytes and vascular smooth muscle cells in the vessel wall (Figure 1) (210–212). Cancer-associated endothelial cells often display an enhanced angiogenic potential (211), due to proangionenic factors secreted from cancer cells and/or tumor–stroma cells, forming heterogeneous neovasculature of enhanced permeability (6). The role of pericytes in tumor vascular development is still largely unexplored (212).

Tumors outgrowing their vascular supply lead to constant vascular remodeling, acute and permanent hypoxic tumor areas, and nutrient deprivation (23, 45, 213), increasing treatment resistance (due to, e.g., reduced radical formation in hypoxic tumor areas affecting radiotherapy and limiting drug delivery of chemotherapeutics) (214–216).

#### Imaging of Tumor Vascularity

Vascular function and distribution has been assessed *in vivo* non-invasively by MRI (45, 217–226), CT (μCT) (45, 52, 227, 228), H_2_^15^O or ^11^C PET (45, 217), US (45, 52, 229) [clinical (230)], PAI (45, 58, 114), and intravital optical imaging methods (231), such as FLI (48), second-generation OCT [optical frequency domain imaging (OFDI)] (Figure 5) (45, 48, 52, 68), and FMT (45).

In vascular MRI, five acquisition methods are used to measure the enhancement of exogenous (i, ii) or endogenous (iii–v) contrast dynamically: (i) Dynamic contrast-enhanced (DCE)-MRI, which exploits the shortening of the T_1_ relaxation time of water protons near a CA, typically Gd-based and low molecular weight (223, 229), (ii) dynamic susceptibility contrast (DSC)-MRI, which measures the effect of the CA (e.g., Gd-based CA or superparamagnetic iron oxide particles) on the T_2_ and T_2_* relaxation time of nearby water protons (218, 222), (iii) arterial spin labeling (ASL)-MRI, where the dynamic measurement of the in- and out-flow of magnetically labeled water protons, which serve as endogenous “CA,” characterizes the vasculature in a region of interest (223, 232), (iv) blood oxygen level dependent MRI (BOLD-MRI) where oxyhemoglobin confers diamagnetic and deoxyhemoglobin paramagnetic contrast, respectively (223), and (v) diffusion-weighted MRI (DW-MRI), where intravoxel incoherent motion (IVIM) reflects vascular perfusion (64, 224, 229, 233, 234).

In DCE- and DSC-MRI, the first pass of the exogenous CA uptake is characterized by the venous or arterial input function (219, 220, 222, 235–237), after which the CA distributes throughout the vasculature, extravasates at sites of leaky blood vessels into the interstitium, and is ultimately cleared from the body. Rate and path (e.g., liver and kidney) of tissue and vascular clearance are dependent on the specific CA (e.g., size and type of CA) used (223, 225, 238). Hemodynamic parameters, such as vessel density, vascular permeability, vascular perfusion, extravascular space, vessel size and plasma volume, etc., are either derived from semiquantitative measures of signal enhancement (220, 239–241) or from pharmacokinetic modeling of signal-*versus*-time curves (222, 242) with the underlying principle based on standard tracer-kinetic theory of linear and stationary systems (221, 243) and the model adjusted to account for variations of CA characteristics, e.g., low versus high molecular weight (impacting the ability of the CA to extravasate), receptor targeted (e.g., α_ν_β_3_-integrin), extra- or intracellular or both, with exchange (218, 223, 235, 244). More recently vascular feature-based analyses have been developed to assess intratumor vascular heterogeneity (245, 246).

Hemodynamic parameters conferred from ASL- and BOLD-MRI are tissue blood flow and volume (223, 232). Tissue perfusion and diffusivity are obtained from IVIM DW-MRI by fitting data with a bi-exponential model (224).

Similarly to DCE-MRI, DCE-CT has been used clinically to obtain blood flow, blood volume, and permeability with the disadvantage of radioactivity limiting serial monitoring and often worse spatial resolution than DCE-MRI (227, 228). In preclinical models, the relatively high radiation dose of μCT limits its *in vivo* use (45), and vascular networks have been assessed *ex vivo* (247). Viscosity of CA for DCE-CT may also result in complications, such as vessel rupture, and DCE-μCT still suffers from artifacts generated from, e.g., bones or large vessels (52). Generally, functional vascular parameters similar to DCE-MRI can be extracted from DCE-μCT data (52, 248).

While H_2_^15^O PET measures specifically tissue perfusion (217), the short half-life of ^15^O limits the applicability of vascular perfusion measurements by PET (45). Vascular permeability can be obtained from dynamic PET using the macromolecular CAs ^68^Gd-DOTA-albumin (45), ^11^C-methylalbumin (limited by the 20.4 min short half-life of ^11^C), or ^68^Ga-transferrin (217). However, lack of accompanying anatomical information and ionizing radiation limits (especially serial) vascularity measurements by PET, despite its potential higher sensitivity than DCE-MRI and its ability to directly measure tissue perfusion.

Tumor angiogenesis, i.e., perfusion and vascular density have been successfully measured in small animals by Doppler or contrast-enhanced US with microbubbles (non-targeted and targeted to, e.g., VEGFR2 (249), α_ν_β_3_-integrin, or endoglin) with a spatial resolution of ~50–100 μm (45, 52, 229). One advantage of the combining contrast-enhanced and non-contrast-enhanced high-frequency volumetric power Doppler US is the ability to distinguish mature and immature vessels (52). While US is cheaper than DCE-MRI, it is not suitable for whole-body imaging, has limited soft tissue contrast, and is to some extent user dependent, limiting its applicability, especially for monitoring of antiangiogenic treatments (52).

In PAI of tumor angiogenesis, the intrinsic contrast from hemoglobin permits visualization of microvasculature and quantification of blood oxygen saturation (45, 52), with its usefulness recently extended to flow imaging (114). Submillimeter resolution is achieved, albeit restricted to depth of a few centimeters (58). Reporter genes or endogenous targeted CAs extend the ability to visualize and quantify tumor angiogenesis *in vivo* (52). The hemodynamic response to external stimuli or treatment is quantified from contrast changes after image reconstruction (52, 58, 114).

Blood vessel diameter, surface area, and branching pattern have been assessed with intravital optical imaging methods during tumor growth and/or treatment (Figure 5) (45, 48, 52, 68, 231).

#### Imaging of Lymphatic Tumor Vasculature

Imaging of the lymphatic system is here summarized only briefly, as is has been reviewed in detail previously (50, 250). The lymphatic system (Figure 1) drains lymph fluid from interstitial space to the venous circulation, thus, maintaining tissue fluid homoeostatis, transports immune cells to lymphoid organs, and plays a role in lipid absorption (50, 250). While the lymphatic vasculature near tumors provides a route for metastatic dissemination of cancer cells, its role has only been explored by non-invasive imaging methods over the past decade (50, 250). Lymphangiography traces the drainage of a CA for X-ray, CT, MRI, US, PAI, or optical imaging but is lacking specificity, requiring direct injection into a lymph vessel (difficult to perform in preclinical models) or intradermal injection near sites draining into the dermal capillary plexus (50, 250). Identifying sentinel lymph nodes containing cancer cells has been achieved with intravenously injected CAs that identify blocked drainage or directly target cancer cells (50). *In vivo* OCT (or OFDI) and laser speckle imaging permit CA-free visualization of lymphatics (Figure 5) (68, 250). The most specific imaging approach to identify and characterize the lymphatic system is to use CAs, targeted to lymphatic vascular-specific molecules, i.e., vascular endothelial growth factor receptor-3 (VEGFR-3), lymphatic vessel hyaluronan receptor-1 (LYVE-1), podoplanin, or prospero-related homeodomain transcription factor PROX1, with LYVE-1 the most widely used lymphatic endothelial cell marker (250). LYVE-1-targeted CAs have been developed for PET (251) and optical imaging (252). Preclinically, fluorescent or bioluminescent gene reporters in transgenic mouse models have been also been used to visualize lymphatics, typically by intravital imaging methods (50).

### Metabolic Imaging

Metabolic reprograming during tumor development and progression leads to a characteristic *in vivo* tumor metabolic phenotype (see Section “Introduction—The Tumor Microenvironment”).

#### Choline-Phospholipid Metabolism

Changes in choline-phospholipid metabolism have typically been assessed preclinically (and clinically) by non-invasive ^31^P and ^1^H magnetic resonance spectroscopy or spectroscopic imaging (MRS or MRSI) (14–16, 18, 253). Choline uptake and metabolic conversion have also been assessed with high sensitivity by ^18^F-fluoro-, ^3^H-, or ^11^C-choline PET and successfully translated to the clinic (254–257). As with any PET tracer though, radiolabeled choline PET does not discriminate between different metabolites (256), limiting its value for pathway studies.

#### Hypoxia

Tumor hypoxia has been imaged non-invasively and visualized directly by hypoxia markers accumulating in hypoxic cells using PET, electron paramagnetic resonance (EPR), or ^19^F MRS (65, 248, 258–266). Delivery of a specific hypoxia marker to less vascularized regions, which are typically associated with hypoxia, may impact the intensity of the accumulating hypoxia marker (246, 267). Since evolution of chronic or acute hypoxia in tissue is linked to the vascular delivery of oxygen, several indirect MRI methods based on vascular features associated with tumor hypoxia, such as BOLD, TOLD, or DCE-MRI, have been developed to identify hypoxic areas or hypoxia changes in tumors (64, 246, 268–271). Tissue oxygen tensions have been mapped by ^19^F MRI oxymetry using perfluorocarbons; as with any exogenous tracer, these measurements are vascular delivery dependent, and thus, potentially biased toward well-perfused tumor regions (271). While carbonic anhydrase-IX (CA-IX) has been proposed as an intrinsic hypoxia marker in tumors, CA-IX expression, measured by immunohistochemistry, correlated to hypoxia in some and not in other studies (260). Nevertheless, attempts are underway to image CA-IX expression *in vivo* by PET (272), NIRF (273), and SPECT (274).

#### Glycolysis and Lactate

Tumor glycolysis is typically assessed by *in vivo* PET using the cellular entrapment of ^18^F-FDG, after uptake of ^18^F-FDG by glucose transporters (GLUT-1, GLUT-3; often overexpressed in cancer) and subsequent phosphorylation by hexokinase II (256, 275). While it is quite insensitive, ^13^C MRS has been applied preclinically to evaluate glycolysis and the ^13^C labeling of downstream metabolites (275–277). Detection sensitivity of ^13^C MRS can be significantly improved by magnetization transfer techniques or indirect inverse detection (276, 277). Hyperpolarization of ^13^C-labeled substrates increases detection sensitivity up to 10,000-fold, with the caveats that the hyperpolarization is short lived and currently limited to few substrates, including glucose (64, 275, 276, 278, 279). Compressed sensing can further improve acquisition speed and spatial resolution in hyperpolarized (HP) ^13^C MRSI (280). Lactate production from precursors, such as ^13^C-labeled pyruvate or glucose, can be rapidly assessed globally and localized by HP ^13^C MRSI (278, 279). Steady state levels of tumor lactate have been assessed by ^1^H MRS and MRSI, using spectral editing methods to suppress the high lipid signal overlapping lactate (64, 275, 281–285).

#### pH

Measuring tumor tissue pH non-invasively is challenging, and various methods have been and are being developed to measure preclinically extracellular and/or intracellular pH (pHe or pHi) (64, 286).

Tumor pHe and pHi distributions can be obtained by ^31^P MRS/MRSI using 3-aminopropylphosphonate (287, 288). However, insensitivity of ^31^P MRSI limits its spatial resolution and restricts broad applicability. Thus, ^1^H MRSI pHe markers have been developed to improve detection sensitivity (288–291). To shorten acquisition times and improve sensitivity further, with potential for clinical translation, HP ^13^C MRSI of injected bicarbonate has been proposed for pH imaging, with pH (predominantly pHi, though it does not distinguish between pHi and pHe) calculated from the signal intensity ratio of hyperpolarized ${\text{H}}^{13}{\text{CO}}_{3}^{-}$ to ^13^CO_2_ (292, 293). However, the reaction is dependent on CA-IX activity in the tissue, thus, calibration has to be performed for each tissue type separately, restricting its applicability (294). Chemical exchange saturation transfer (CEST) MRI detects the pH-dependent chemical exchange between an amide proton and surrounding water molecules (291, 295). In acidoCEST, the pH dependence of the CEST effect ratio of a CEST agent with two amide protons, generating two CEST effects, is used to measure pHe (295–297). Challenges to measure pH by acidoCEST include its low sensitivity, requiring optimization of experimental parameters (295), and it may not always be a given that the CEST effect is solely visible for the amide protons of the selected CEST agent and only affected by pH (291).

Recently, the pH-dependency of cellular membrane insertion of radiolabeled pH (low) insertion peptides has been used to image tumor pH (at the intra-/extracellular interface) preclinically with PET (298–301).

Other non-invasive imaging modalities assessing pH *in vivo* include optical imaging with pH-sensitive dyes (302–304) or a pH-sensitive reporter gene (305), ratiometric PAI with pH-sensitive nanoprobes (306, 307), MRI using a CA with pH-sensitive (and concentration-dependent) relaxivity, with the difficulty of measuring *in vivo* the CA concentration (291), and EPR spectroscopy (308, 309).

Additionally, pH-sensitive probes are being developed as theranostic agents, combining treatment with diagnostic and monitoring ability (310–314). Of note is that all exogenous pH markers are delivery dependent and may not be clinically translatable, adding further challenges to pHe/pHi imaging.

## Non-Invasive Multimodal Imaging of Tumor–Stroma Interaction

Here, after a brief overview and some examples of recently recognized tumor–stroma interactions (see Section “Tumor–Stroma Interactions”), ongoing efforts to apply directly non-invasive multimodal imaging to characterize and understand tumor–stroma interaction in the context of tumor development, progression, and treatment will be summarized (see Section “Non-invasive *In Vivo* Imaging of Tumor–Stroma Interactions”). As is evident from the comparably fewer studies (see Section “Non-invasive *In Vivo* Imaging of Tumor–Stroma Interactions”), it is much more challenging to image directly and non-invasively the tumor–stroma interaction in *in vivo* cancer animal models (237, 315) and to confirm *in vitro* and *ex vivo* findings.

### Tumor–Stroma Interactions

Tumor stroma interactions focus on the complex crosstalk between cancer and stromal cells and cell interactions with the ECM (316–320). These interactions are mediated by chemokines, soluble factors from enzymes, growth factors, extracellular vesicles (e.g., exosomes) and/or microRNAs, etc., and regulate enzymes activities, expression of genes and proteins, and metabolic pathways involved in tumor growth, metastases, survival, and drug resistance (37, 188, 211, 319–325). In this section, we present selected examples of *in vitro, ex vivo*, and *in vivo* tumor growth studies that highlight tumor–stroma interactions by using preclinical models that attempt to incorporate/simulate microenvironmental conditions of ultimately clinical relevance.

Various *in vitro* models mimicking the TME, such as cocultures between stromal and tumor cells or CAF-derived exosomes and cancer cells (326), 3D culture systems (327), bioreactors for live cell studies (20, 277, 328–330) have been developed to understand the nature and mechanisms behind tumor–stroma interactions by, e.g., gene expression microarrays from cocultures (331).

For example, in *in vitro* 2D and 3D cultures of the two breast cancer cell lines MDA-MB-231 and MCF-7 cocultured with CAFs or control fibroblasts, CAFs promoted invasion and proliferation in both MDA-MB-231 and MCF-7, and the more invasive MDA-MB-231 increased α-smooth muscle actin (α-SMA, a marker of fibroblast-to-myofibroblast transition) expression of CAFs contrary to the non-invasive MCF-7 (332), demonstrating reciprocal interaction. In cocultures of the cervical cancer cell line CSCC7 with CAFs or control fibroblasts, increased CSCC7 migration was associated with a CAF-induced decrease and partial replacement of fibrillar ECM components with laminin-1 (148). In 3D cocultures of oral tongue squamous cancer cells and CAFs in matrigel, CAFs (and CAF-conditioned medium) promoted growth, proliferation, migration, and epithelial-to-mesenchymal transition of the cancer cells (333). As observed by OCT, 3D cocultures of breast cancer cells and immortalized fibroblasts induced larger and more spherical acini with increased lumen size than cocultures using immortalized breast cells (101). Besides CAFs, the presence of TAMs has been shown also to affect ECM remodeling (334). For example, excretion of MMPs into the supernatant increased significantly in coculture of two breast cancer cell lines and macrophages, enhancing tumor cell invasiveness, and not in the benign breast cell line/macrophage coculture (335).

Tumor cells and CAFs also interact metabolically (Figure 2). As shown *in vitro*, CAFs take up and metabolize extracellular lactate (38) and export pyruvate which is taken up and metabolized by cancer cells (336) (Figure 2). Other research implies that epithelial cancer cells use metabolites, such as lactate, ketone bodies, and glutamine, excreted by CAFs in response to cancer cell-induced oxidative stress (39, 334, 337) (Figures 1 and 2). Glycolysis and glutamine-dependent reductive carboxylation increased in cancer cells following oxidative phosphorylation (OXPHOS) inhibition induced by exposure to CAF-derived exosomes (326). Additionally, immune cells and adipocytes may further impact the metabolic tumor phenotype (334). Closer to the *in vivo* scenario, *ex vivo* tumor/stroma immunostaining, molecular profiling from tissue microarrays of excised tumors (338), or multiplexed staining and *in situ* transcriptome profiling techniques (339) improve further our understanding of tumor–stroma interaction. For example, Choi et al. (338) classified breast cancer subtypes of patient tumors into four subgroups defined by the *ex vivo* expression of the glycolysis markers Glut-1 and/or CA-IX in the tumor and stroma, respectively: Warburg type (tumor: GLUT-1 and/or CA-IX positive; stroma: Glut-1 and CAIX negative), reverse Warburg type (tumor: Glut-1 and CAIX negative; stroma: GLUT-1 and/or CA-IX positive), mixed type (tumor and stroma: GLUT-1 and/or CA-IX positive), and null type (tumor and stroma: Glut-1 and CAIX negative). The Warburg and mixed type were predominantly associated with triple-negative breast cancer, while the reverse Warburg and null-types predominantly associated with luminal breast cancer (338).

These data/models of metabolic interaction between cancer cells and CAFs or other stromal cells highlight the complexities of metabolic crosstalk and the need for further research to understand how metabolic plasticity of tumor and stromal cells benefit tumor progression and evasion of treatment.

While MSCs can dedifferentiate into various stromal cells after recruitment to tumors, many questions about the mechanisms of MSC homing and MSC–cancer cell interaction are still topics for future research (155). In a recent study, MSCs promoted *in vivo* growth of subcutaneous colorectal tumor models by a β1-integrin-dependent interaction of MSCs and cancer cells (340). Coinjection of breast or prostate cancer cells with either normal fibroblasts or CAFs into animal models showed that, compared to normal fibroblasts, the presence of CAFs enhanced tumor growth (341, 342) and, as shown for the breast model, increased angiogenesis through elevated stromal cell-derived factor 1 *via* recruitment of endothelial progenitor cells (341). As demonstrated by *in vivo* fluorescence imaging and caliper tumor volume measurements, coinjection of human endometrial cancer cells with CAFs into nude mice increased tumor growth compared to tumor initiation without coinjection of CAFs (343). It was shown that the proliferation of endometrial cancer cells was increased in the presence of CAFs through the activation of JAK/STAT3/c-myc pathway (343).

Tumor–stroma interactions may sensitize tumors to treatment or be a source of treatment resistance across a wide range of therapeutics (320). And targeting tumor–stroma interactions by targeting its mediators, such as chemokines, may improve treatment response. For example, as observed with BLI, treatment of a prostate cancer model with the CXCR4-specific inhibitor AMD3100 in combination with docetaxel significantly reduced tumor growth compared to docetaxel alone (344). As a high-throughput alternative to *in vivo* models, an *in vitro* tumor cell-specific bioluminescence imaging (CS-BLI) assay for tumor–stroma cell cocultures has been proposed (345). Using this assay, it was shown that multiple myeloma cells exhibited chemoresistance to dexamethasone and doxorubicin when cocultured with bone marrow stromal cells, while effectiveness of reversine was enhanced by the presence of stromal cells (345).

Novel treatments, targeting tumor–stroma interaction by therapeutic targeting of adhesion, proteolysis, and/or signaling pathways, may improve on current treatment regimens and overcome treatment resistance (2, 346).

### Non-Invasive *In Vivo* Imaging of Tumor–Stroma Interactions

Studying tumor–stroma interactions *in vivo* enables the comprehensive characterization of the TME and its impact on treatment efficacy, potentially leading to improved diagnosis, to the identification of new treatment targets, and closing further the gap between preclinical and clinical studies (320). While single imaging methods have been used to image different aspects of the TME, only recently multimodal imaging has become more frequent. One major challenge of imaging the TME is that tumor and stromal cells use common pathways (286), necessitating cell-type-specific labeling and ideally imaging with cellular resolution. Localized, high-resolution imaging or combining multiple imaging modalities may to some extent overcome this inherent challenge. Intravital microscopy (66), which is considered a minimally invasive imaging modality, provides high-resolution imaging, including imaging of cellular processes (86, 208, 347), and has been to date the method of choice to study cancer cell interaction with the TME (Figures 3–5).

By using transgenic mice expressing green fluorescent protein (GFP) in all cells or in specific organs or driven by a cell marker and tumor cells expressing red fluorescent protein (RFP) (237, 348) or GFP in the nucleus and RFP in the cytoplasm (349), whole-body fluorescence imaging has been used to study tumor–TME interactions. While morphology and location of cells may help to identify what type of stromal cell may be involved in a specific biological process (237), specificity is lacking as all cell types of the host express the same fluorescence and *ex vivo* studies are needed for confirmation (349). By *ex vivo* validation of cell types, it was confirmed in a GFP-expressing mammary tumor model and a host with GFP-expressing macrophages, that both, cancer cells and macrophages migrated into microneedles filled with EGF, TGF-alpha, and CSF-1, as detected by multiphoton intravital microscopy (350). Using these techniques, it has been shown that paracrine loops associated with macrophage and tumor cell interaction impact tumor cell migration, intravasation, and dissemination (351).

Using intravital microscopy with multiphoton laser scanning microcopy (LSM) and SHG imaging of a human soft tissue sarcoma in VEGF-GFP mice, increased ECM remodeling by CAFs after exposure to relaxin has been imaged *in vivo*, with the involvement of CAFs confirmed by *ex vivo* cell typing (352). Using human, DsRed2- and nuclear histone 2B (H2B)-EGFP-expressing fibrosarcoma cells implanted into deep dermis of nude mice, tumor growth and tumor cell invasion into the surrounding tissue could be imaged by epifluorescence microscopy (Figure 3A) (66). In the same tumors, morphology (including collagen fibers), neoangiogenesis, cancer cell mitosis, and apoptosis were assessed *in vivo* during tumor growth by intravital microscopy with FLI and SHG (Figure 3B) (66). Multiphoton LSM combined with collagen (SHG) imaging of murine mammary tumors grown from a mix of a low-metastatic cell line expressing GFP and a high-metastatic subline transfected expressing CFP (cyan fluorescence protein) in the cytoplasm has been used to track and visualize cell shape, subcellular structures, and behavior *in vivo* (67) (Figure 4). The motility of the cells with the larger metastatic potential was about 4.5-fold higher than in the cells with low-metastatic potential with migration along collagen fibers (67).

Beyond migration and imaging of vasculature and collagen structures, the redox ratio based on endogenous NADH/(FAD + NADH) had been imaged by intravital microscopy with multiphoton fluorescence lifetime microscopy (FLIM), and redox ratio changes have been found to relate to changes observed by ^18^F-FDG PET, and, in ovarian cancer, were related to disease risk (49). As fluorescence lifetime changes with binding state and TME of metabolic enzymes (49), multiphoton FLIM, combined with other imaging modalities and intravital microscopy, is uniquely qualified to observe such changes *in vivo*, with the limitation of imaging depth.

Tumor vascularization, lymph vasculature, and vascular response to treatment have also been evaluated by intravital microscopy within the context of tumor growth and collagen structures (Figure 5) (66, 68, 86). Alexander et al. (66) imaged the intra- and perilymphatic invasion of fluorescent fibrosarcoma cells, indicative of a potential route of metastatic dissemination *via* the lymph vasculature located at the tumor margin (Figure 5A). Using intrinsic contrast, Vakoc et al. (68) imaged the antivascular effect of an antiangiogenic agent inhibiting VEGFR-2 on the tumor vasculature *in vivo* at the microscopic level, depicting lymph and blood vessels (Figure 5B). They found in response to VEGFR-2 blockade that intratumor vessel length and mean vessel diameter decreased, as tumor growth was delayed (68).

Nakasone et al. (353, 354) showed by intravital microscopy with a microlensed spinning-disk confocal microscope (355) of tumors in MMTV-PyMT mice expressing ACTB-ECFP in all host cells and c-fms-EGFP in myeloid cells, respectively, that vascular permeability and innate immune cell infiltration impact response to doxorubicin. The accumulation of macrophages with tumor growth as well as increased macrophage infiltration with increased metastatic ability have been imaged non-invasively in breast cancer models by fluorescence-reflectance imaging using a fluorescently labeled specific probe for alarmin S100A9, a calcium-binding protein secreted by monocytes/macrophages with the protein complex S100A8/A9 acting as mediator between tumor and immune cells (69) (Figure 6).

The fairly recent development of MRI/PET instrumentation permits the simultaneous imaging of metabolic, anatomical, and dynamic information, including cell tracking using appropriate labeled probes, during tumor progression and in response to treatment (70, 356–358) (Figure 7). The ability to effectively observe intratumoral function and heterogeneity over time by simultaneous MRI/PET has been demonstrated in a carcinoembryonic antigen-expressing colorectal adenocarcinoma model (Figure 7) (70). A recent study showed that microvessel volume and density index (determined from MRI) were significantly lower for glioblastoma tumors treated with bevacicumab and the PI3K/mTOR inhibitor BEZ235 combined than for tumors treated with bevacicumab alone, while ^18^F-FET (O-(2-[^18^F]Fluoroethyl)-l-tyrosine) uptake, a PET tracer to assess vessel amino acid transport, remained unchanged between the two treatments (359). Further, tumor growth, as determined from MRI, and cell proliferation, as determined from ^18^F-FLT PET, were the same for the bevacicumab/BEZ235 combination therapy and the bevacicumab alone treatment groups (359). The *in vivo* results were validated by *ex vivo* studies (359).

While still significant more research needs to be done, these studies show the potential of harnessing the strengths of different imaging modalities to image tumor–stroma interaction within the TME *in vivo*, and thus, enhancing our understanding of its impact on tumor growth and treatment response.

## Conclusion

The strengths of optical imaging are its high sensitivity for CAs, ability to use a wide range of probes, including activatable probes and reporter genes, and compared to other imaging modalities, such as MRI and PET, low cost. However, optical imaging is typically semiquantitative, limited by penetration depth, small field of view, and, depending on method, high background signals and lack of tomographic information. Some of these limitations are overcome by PAI, which permits real-time quantitative imaging but is hampered by the range of available CAs. Ultrasound imaging is a low cost, rapid, real-time imaging modality with high temporal and spatial resolution, but has a limited field of view with low soft tissue contrast, and is typically semiquantitative and user dependent. Computer tomography is rapid, permits whole-body imaging, has high spatial resolution, is user independent, and mostly low cost, but is limited by its low sensitivity to CAs, lack of endogenous soft tissue contrast and exposure to radiation. Scanners for MRI (MRSI), PET, and SPECT are high in cost with the distinct advantage of whole-body imaging capabilities. While MRI has excellent soft tissue contrast, high spatial resolution, and has a wide range of methods available for tissue imaging and vessel characterization, it is limited by its low sensitivity and the fairly long acquisitions, the latter particularly prominent in spectroscopic imaging. To overcome these challenges, new methods, such as hyperpolarized ^13^C MRSI, are being actively developed. The high sensitivity of PET and SPECT, respectively, is offset by their low resolution (1–2 mm), lack of morphological information and radiation exposure from the radioactive tracers, whose half-lives range from 75 s (Rb-82) to 4.18 days (I-124) for PET radioisotopes and from 6 h (Tc-99m) to 59 days (I-125) for SPECT tracers. With the advancement of MRI/PET, the power of various MRS and MRI methods beyond anatomy and DCE-MRI, such as MRS(I), can be harnessed for future studies, distinguishing itself from PET/CT with reduced radiation exposure, the latter making MRI/PET a powerful tool for serial monitoring.

While CT, MRI (MRSI), PET, SPECT, and US are already standard imaging tools in the clinic, for localized applications, e.g., detecting cancer cells at tumor margins during surgery (360), optical imaging is being assiduously developed. Aside from physical parameters specific to each imaging modality, clinical imaging of tumor–stroma interaction will also be in part defined by the successful development of safe tracers/CAs.

In the majority of preclinical studies, specific aspects of the TME and its stromal components have been investigated separately (a few aspects at a time), selecting the non-invasive preclinical imaging modality best suited for the task. However, recent strong evidence pointing to the importance of the interaction between tumor cells and multiple components of the TME in tumor development, growth, metastases, and treatment response, including drug resistance, has generated a strong interest to further develop imaging technologies to investigate tumor–stroma interactions non-invasively *in vivo*. Despite recent research efforts, the comprehensive characterization (including serial monitoring) of the TME and tumor–stroma interactions non-invasively *in vivo* requires further advancement and to take advantage of the strengths of multimodal imaging tools for preclinical studies, and ultimately for clinical translation.

## Author Contributions

EA and NR: conception, design, and writing of review article.

## Conflict of Interest Statement

The authors declare that the research was conducted in the absence of any commercial or financial relationships that could be construed as a potential conflict of interest.
